# Single-Stage Microfluidic Synthesis Route for BaGdF_5_:Tb^3+^-Based Nanocomposite Materials: Synthesis, Characterization and Biodistribution

**DOI:** 10.3390/ijms242417159

**Published:** 2023-12-05

**Authors:** Zaira Gadzhimagomedova, Ilia Pankin, Vladimir Polyakov, Darya Khodakova, Pavel Medvedev, Pavel Zelenikhin, Nail Shamsutdinov, Sergey Chapek, Anna Goncharova, Alexander Soldatov

**Affiliations:** 1 The Smart Materials Research Institute, Southern Federal University, 344090 Rostov-on-Don, Russia; zgad@sfedu.ru (Z.G.); pmedvedev@sfedu.ru (P.M.); chapek@sfedu.ru (S.C.); soldatov@sfedu.ru (A.S.); 2 National Medical Research Centre for Oncology, 344037 Rostov-on-Don, Russia; hodakovadv@rnioi.ru (D.K.); goncharovaas@rnioi.ru (A.G.); 3 Institute of Fundamental Medicine and Biology, Kazan Federal University, 420008 Kazan, Russia; pavel.zelenikhin@kpfu.ru (P.Z.); michaeldermoon@gmail.com (N.S.)

**Keywords:** nanoparticles, nanocomposites, X-ray photodynamic therapy, microfluidic, nanophosphor, photosensitizer, computed tomography, biodistribution

## Abstract

Rare-earth-doped nanoscaled BaGdF_5_ is known as an efficient contrasting agent for X-ray micro-CT and NMR as well as a promising candidate for X-ray photodynamic therapy, thereby opening an opportunity for theragnostic applications. Conventional synthesis of Ln-doped BaGdF_5_ consider a long-lasting batch procedure, while a conjugation with photosensitizer usually implies a separate stage requiring active mixing. To the best of our knowledge, in this work, we for the first time obtain BaGdF_5_:Tb^3+^ nanophosphors in a microfluidic route at temperatures as low as 100 °C while decreasing the time of thermal treatment down to 6 min. The proposed synthesis route allows for the obtaining of single-phase and monodisperse BaGd_1−x_F5:Tb_x_^3+^ nanoparticles with an averaged particle size of ca. 7–9 nm and hydrodynamic radius around 22 nm, as estimated from TEM and DLS, respectively. In addition, X-ray-excited optical luminescence has been recorded in situ for the series of nanophosphors synthesis with varied flow rates of Tb^3+^ and Gd^3+^ stock solutions, thereby anticipating a possible application of microfluidics for screening a wide range of possible co-dopants and reaction conditions and its effect on the optical properties of the synthesized materials. Moreover, we demonstrated that BaGd_1−x_F_5_:Tb_x_^3+^@RoseBengal conjugates might be obtained in a single-stage route by implementing an additional mixer at the synthesis outcome, namely, by mixing the resulting reaction mixture containing nanoparticles with an equivalent flow of photosensitizer aqueous solution. In vitro cytotoxicity test declares moderate toxicity effect on different cell lines, while the results of flow cytometry indirectly confirm cellular uptake. Finally, we report long-term biodistribution monitoring of the synthesized nanocomposites assessed by X-ray micro-CT in the in vivo experiments on balb/c mice, which depicts an unusual character of agents’ accumulation.

## 1. Introduction

Photodynamic therapy has long been known as an effective method for treating surface cancer tissues [1,2]. Although the approach is widely used in modern medicine, due to several limitations, it cannot be implemented efficiently for the non-invasive treatment of deep-localized pathologies and tumors. Recently, X-ray photodynamic therapy (XPDT) has been proposed as an efficient alternative to conventional radiotherapy of cancer, which typically requires precise focusing of the X- or Gamma-rays and relatively high exposure of the patient [3,4,5,6,7]. However, at the same time, XPDT requires efficient and biocompatible composite materials, which typically consist of two components—nanophosphors capable of re-emitting X-rays into visible light that, in turn, is absorbed by photosensitizer responsible for the generation of reactive oxygen species (ROS), which cause cell death.

Fluorides of heavy elements, such as Ba, Gd and La, doped with rare-earth elements (REE) are among the most promising nanophosphors as a part of the nanocomposite materials for XPDT [8,9,10]. Their advantages include resistance to X-ray irradiation, chemical stability, efficient energy transfer from the host matrix to the doping elements, and the ability to fine-tune luminescence by varying the type and concentration of REE doping [11,12].

The morphology, particle size, and luminescent properties of BaGdF_5_ nanophosphors doped with different rare-earth elements have been previously discussed in refs [13,14,15,16,17,18,19,20]. In particular, D. Yang et al. have reported sub-10 nm BaGdF_5_:Yb^3+^/Tm^3+^ up-conversion nanoparticles (NPs) synthesized at 300 °C, stabilized by oleic acid and subsequently loaded by doxorubicin to enhance its anti-cancer activity [17]. BaGdF_5_ doped with Yb^3+^ and Ho^3+^ up- and down-conversion solid solutions has been synthesized via a facile additive-assisted hydrothermal route with a thermal treatment of reaction mixture at 180 °C for 24 h [21]. Guan and co-workers have reported an autoclave hydrothermal synthesis with a temperature treatment of 180 °C for obtaining Dy^3+^/Tb^3+^/Eu^3+^ -doped BaGdF_5_ nanoparticles with a spherical shape and averaged size around 20 nm [22]. Becerro et al. have proposed surfactant-free synthesis based on homogeneous precipitation using Ba, Gd and RE acetylacetonates as a precursor that allows for the release of cations in a reaction medium in a controlled manner [23]. The reported approach allows for obtaining of hydrophilic uniform BaGdF_5_ with tunable size (controlled by Ba and Gd precursor concentrations) in the range from 45 to 85 nm, which were obtained at temperatures as low as 120 °C and an aging time of 10 h. More recently, W. Liu and co-workers have reported a hydrothermal synthesis of BaGdF_5_:RE microcrystals and sub-micron particles of octahedral shape, where environmentally friendly ionic liquid 1-octyl-3-methylimidiazolium tetrafluoroborate ([Omim]BF_4_) were employed as structure-driven agent [24]. Moreover, due to efficient, fast, and homogeneous heat transfer, microwave synthesis has been implemented for lanthanide fluoride synthesis [25,26,27]. In particular, Y. Lei and co-workers [28] have reported a microwave-assisted synthesis of fluorescent hydrophilic nanocrystals with BaYF_5_ host matrix, while our group has previously implemented microwave treatment at 200 °C for obtaining Tb, Eu, and Sm-doped BaGdF_5_ nanoparticles with a narrow size distribution [20].

As mentioned above, hydro/solvothermal and microwave synthesis of BaGdF_5_:Ln nanophosphors are generally characterized by the relatively high temperature and long duration of thermal treatment, as well as, in most cases require the use of PTFE (Teflon) autoclave or tightly closed vessels, which makes the synthesis conditions optimization task time and reagent consuming. In spite of the several limitations (common for inorganic objects) discussed in this work, the application of microfluidic for the synthesis of heavy-element fluoride nanophosphors opens up the possibility of rapid screening of different synthesis conditions such as synthesis temperature, solvent composition, and variation in doping element percentages. One of the main advantages of microfluidic flow-through synthesis systems is the possibility of more efficient mixing of the reagents in essentially shortened time, screening of a wide range of synthesis parameters with moderate time and reagent consumption, as well as an efficient heat transfer without undesirable temperature gradients thanks to the small cross-section of microfluidic channel and pipes.

Despite the fact that the first synthesis of Ce- and Tb-doped LaF_3_ nanocrystals via microfluidic reactor was reported in 2008 [29], only very few examples of MF synthesis application for similar objects can be found in the literature. Later, X. Zhu and co-workers combined a microfluidic reactor with a channel cross-section of 300 μm and microwave treatment to synthesize water-soluble LaF_3_ nanoparticles and LaPO_4_ nanorods [30]. Recently, D. Liu et al. have reported the synthesis of α-phase NaYF_4_:Tb,Er nanoparticles by using a quartz-tube flow-through setup with an inner diameter of 1 mm in the temperature range from 155 to 255 °C [16]. J. Ma et al. have demonstrated that both the synthesis of Ln^3+^-doped KGdF_4_ nanoparticles and its further surface functionalization by hyaluronic acid can be performed on the microfluidic platform with a simple architecture [31]. Finally, very recently, M. Isikawa and E. Guidelli reported a microfluidic synthesis of GdF_3_:Eu near-infrared scintillation nanoparticles, which surfaces were subsequently modified by growing PAA and Methylene Blue layered structure turning the system into nanocomposite with an efficient ROS generation and linear dose-rate response.

To the best of our knowledge, in the following, we, for the first time, report the microfluidic synthesis of Tb^3+^-doped BaGdF_5_ nanophosphors with the possibility of in situ monitoring of the X-ray-excited luminesce (as a target property) as well as a simple single-stage flow-through microfluidic procedure for obtaining BaGdF_5_:Tb@RoseBengal conjugates accompanied with the report on its biocompatibility and biodistribution.

## 2. Results and Discussion

It is known that conventional batch solvothermal (ST) [32,33,34], as well as microwave (MW) BaGdF_5_ syntheses [20,35], lead to the formation of spherical or irregularly shaped nanoparticles. Moreover, as reported in refs [23,35], the size and morphology of the BaGdF_5_:Ln nanoparticles depend on the solvent composition, which can significantly affect ion diffusion involved in the nucleation and growth process of the particles as well as it can affect surface charge, thus controlling the possible unwanted particle aggregation. Bacerro et al. demonstrated that solvothermal synthesis performed using ethylene glycol or diethylene glycol as a solvent instead of glycerol led to the formation of polydisperse BaGdF_5_ nanospheres. On the other hand, in our recent work [35], where microwave synthesis was applied in pure ethylene glycol NPs with an average size of 10–15 nm were obtained. Based on our experience, adding water to EG leads to an increase in the average particle size in MW synthesis to 20–30 nm and to 40–50 nm in ST synthesis. This effect is due to a decrease in the viscosity of the reaction mixture and acceleration of the NPs growth stage. In addition, the typical synthesis of BaGdF_5_-based nanophosphors proceeds at 200 ℃ for a relatively long time: 24 h in ST conditions and 1–2 h in MW. It is worth noting that the heating of the reaction mixture in ST batch synthesis is uneven and might result in the formation of GdF_3_ as a side product, which might be undesirable for biomedical applications, where obtaining monodisperse colloidal solutions with controlled particle size distribution is of crucial importance [36,37].

### 2.1. Adapting Reaction Conditions in MF Mode

**Figure 1:**
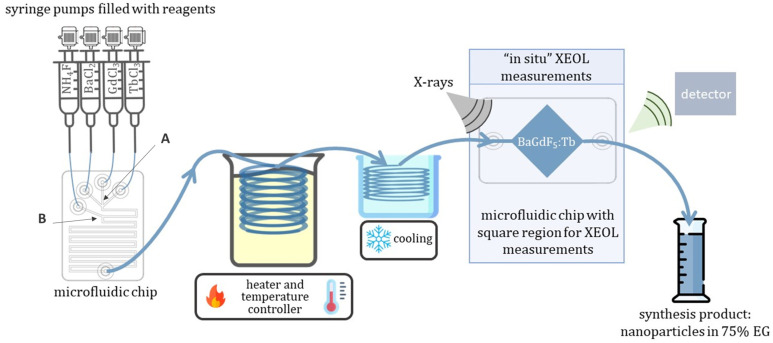
Scheme of experimental setup employed for microfluidic synthesis of BaGd_1−x_F_5_: Tb_x_^3+^ with an in situ XEOL acquisition. A—specify the first mixing point, where precursor solutions of BaCl_2_, GdCl_3_ and TbCl_3_ firstly mixed with each other, while B—specify secondary mixing point, where solution containing NH_4_F enter mixed with other reagents.

To adapt the synthesis conditions in the microfluidic mode, we varied the solvent composition, synthesis time (controlled by the total flow rate of the reagents), and precursor concentration. The main conclusion over the tested reaction condition might be summarized as follows: an increase in the precursors’ concentration, as well as a decrease in the total flow rate and viscosity, lead to clogging of the microfluidic chip (see Figure S1) around the mixing point B (see Figure 1), where ammonium fluoride was added to the system, and a fast and uncontrolled growing of metal fluorides crystals occurred further blocking the flow.

Microfluidic synthesis in pure ethylene glycol (100 EG) showed a low concentration of particles in the resulting solution (~1.5–2 mg/mL, thus resulting in a very low product yield) with an average particle size of 10 nm, according to the quantitative analysis of TEM images. This might be due to the dramatic decrease in reaction rate in a viscous medium. To reduce the viscosity, ethylene glycol was diluted with DI water to the compositions 75%, 50%, and 25% of EG in DI water and DI water only (0% of EG). It was found that the composition of 75EG and 0.1 M solutions of reactants allowed for the obtaining of nanoparticles with a relatively high concentration of 10–12 mg/mL, as was estimated after washing and drying the synthesis products. Starting from 50EG and further, rapid chip clogging occurred due to a sharp increase in the reaction rate followed by fast BaGdF_5_ crystallization on the roughness of the channel’s inner walls (see Table 1). Thus, 75% of EG and 25% DI solution was selected as an optimal medium for microfluidic synthesis. In addition, 75EG solution appears as a favorable condition for the in situ XEOL data collection for the series of synthesis with different numbers of Tb, providing a more homogeneous distribution of reaction mixture (as demonstrated in Figure S13) inside the reservoir of the XEOL chip (see Section 3).

Experiments with different synthesis times were conducted by varying the total flow rates corresponding to 2 h (1.54 μL/s), 1 h (3.08 μL/s), 30 min (6.17 μL/s), 12 min (15.42 μL/s), and 6 min (30.84 μL/s) for the passage time of a volume element of reaction mixture through the entire synthesis setup (where significant part of total pipes lengths belonged to thermal treatment region in oil bath; see Figure 1 below). The obtained particle concentrations are reported in Table 1. Moreover, it was shown that the significant reduction in synthesis time does not spoil the crystallinity of the formed nanoparticles, as revealed by XRD (see Figure S2) for different synthesis times, thus demonstrating that the thermal treatment at 100 °C as short as 6 min was enough for particle growth and formation of MF mode. A dramatic decrease in the synthesis time for MF synthesis might be explained by the fact that the seed formation likely readily occurs right after the initial intensive mixing of all four reagents inside the microfluidic chip, even at RT (which is confirmed by chip clogging at low percentages of EG/slow rates of reagents supply/high concentration of precursor solutions, see Table 1), which is further accompanied by the nanoparticle growth stage with fast and efficient heat transfer inside a PTFE tube with relatively small internal diameter of 2 mm.

Notably, Y. Lei and co-workers [28] have reported ultra-fast (10 min) microwave synthesis for BaYF_5_:Tb/Ce, Tb colloid nanocrystals with high product yield. In our case, when we consider MW synthesis with a duration of 6 min and 0.1 M concentration of metal salt precursors, the concentration of NPs in the resulting synthesis mixture was about 5 mg/mL, while for MF synthesis with similar conditions, the obtained NP concentrations in the synthesis product were in the range of 10–12 mg/mL (as reported in Table 1), thus demonstrating even higher product yield.

Finally, for in situ XEOL characterization reported below, it is important to achieve the maximum possible concentration of the BaGdF_5_:Tb NPs in a stable colloidal solution in order to enhance the luminescence signal. For this purpose, the concentration of metal chloride solutions was varied in the range 0.1, 0.2, 0.3, 0.4, and 0.5 M, while for each synthesis, the concentration of fluoride source NH_4_F was scaled proportionally. The obtained synthesis results demonstrated that concentrations of metal chlorides above 0.3 M led to rapid (5–7 min) microfluidic chip clogging, while for 0.1 M, 0.2 M, and 0.3 M attempts, the concentration of NPs produced in the synthesis increased proportionally with the increase in the precursor’s concentrations (see Table 1).

### 2.2. Synthesis of BaGd_1−x_F_5_:Tb_x_^3+^ Nanophosphors with In-Situ XEOL Acquisition

Within the MF synthesis concept, it is useful to have the ability to monitor the targeted properties of the synthesized nanophosphors in situ. For this purpose, syntheses were carried out with the ability to monitor XEOL spectra from the particle flow, as described below.

The scheme of MF setup for the nanophosphor’s synthesis and in situ XEOL measurements is shown in Figure 1, while the photo of the experimental setup used in this work is reported in Figure S3. A 0.3 M solutions of BaCl_2_·2H_2_O, GdCl_3_·6H_2_O, TbCl_3_·6H_2_O, and 1.65 M solution of NH_4_F were prepared with a content of 75% EG and 25% of DI water. Next, homemade microfluidic setups (MF-1, see description in Section 3) were charged with prepared solutions. The flow rate of each component was controlled by the dedicated homemade software (Smart Fluidics v.1.0.1).

The sum of flow rates for the Gd^3+^ and Tb^3+^ solutions was always kept equal to the flow rates of Ba^2+^ and F^−^ (see Table 2). At the first stage the chloride solutions of Gd^3+^, Tb^3+^, and Ba^2+^ were mixed (at the mixing point A, see Figure 1) and spread along the first bend of a microfluidic reactor, and a solution of ammonium fluoride was then added to the mixture (at the mixing point B at Figure 1). Afterward, the obtained mixture of all four reagents passed through the residual part of the meander passive mixer, maintaining the total flow rate of 30.84 μL/s. Next, the reaction mixture entered a PTFE tube with a total length of 1.5 m and internal diameter of 2 mm, immersed in a silicon oil bath, and kept at a constant temperature of 100 °C (the temperature of the bath was continuously controlled). After the thermal treatment region, the resulting reaction mixture flow inside silicone pipes (internal diameter D_int_ = 1 mm) passing through a cooling bath kept at RT were subsequently sent to the second microfluidic chip dedicated to in situ XEOL signal acquisition (as demonstrated in Figure 1), and finally, synthesis products were collected in a glass/plastic flask. After the synthesis, the obtained reaction mixture solution was collected, centrifuged at 15,000 rmp for 5 min, and thoroughly washed with DI water three times. Then, the obtained precipitate was dissolved in distilled water, forming a stable colloidal solution of nanoparticles. A fraction of 2 mL of each synthesis has been dried to estimate the actual concentration of NPs in the resulting colloidal solutions, as well as for phase and element composition analysis by means of XRD and XRF, respectively.

**Figure 2:**
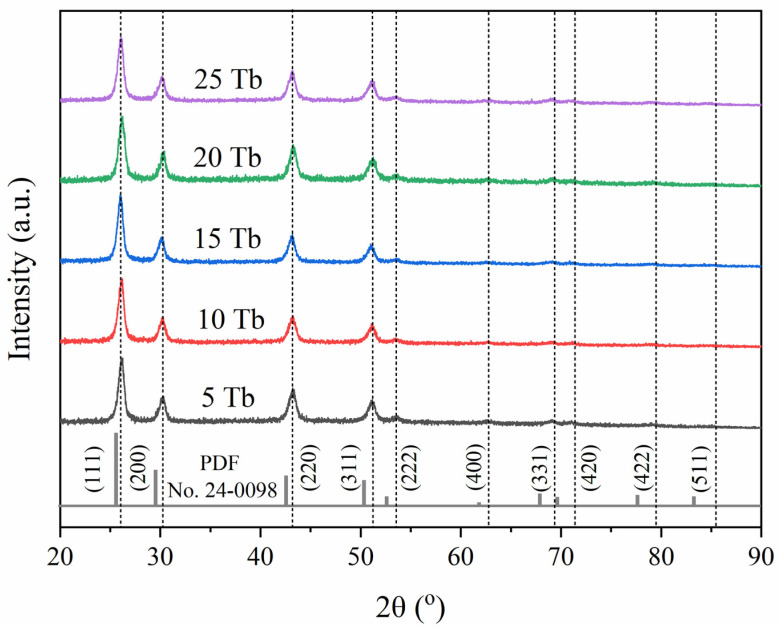
XRD patterns of the BaGd_1−x_F_5_:_x_Tb^3+^ samples synthesized by microfluidic route in comparison with BaGdF_5_ patterns from JCPDS database no. 24-0098.

By varying the flow rates of Gd^3+^ and Tb^3+^ at constant rates of Ba^2+^ and F^−^ (see Table 2), samples containing different percentages of terbium were obtained. Powder X-ray diffraction analysis for the patterns collected on BaGd_1−x_F_5_:Tb_x_ samples (see Figure 2) demonstrated that all synthesized samples with different amounts of doping elements had similar diffraction profiles corresponding to the cubic space group Fm-3m (225) (JCPDS card no. 24-0098 [38]). The profile parameters were refined using the pseudo-Voigt function, as implemented in the Jana2006 program package (version 25/1-/2015) [39] and summarized in Table 3 and Figure S4. The BaGdF_5_ matrix doping with Tb led to the tiny modification of the cell parameters. This can be clearly observed by the gradual shift in diffraction patterns. According to the XRD analysis (see Table 3), the cell parameter is smaller than the PDF database value (a = 6.023 Å). This phenomenon of crystal lattice compression is associated with the size effect typically observed for nanoparticles.

The gradual decrease in the cell parameters and volume of the unit cell upon the increase in doping element concentration with slightly smaller ionic radius (1.063 Å and 1.078 Å, for Tb^3+^ and Gd^3+^, respectively) [40] revealed that doping Tb ions substitute Gd in their lattice positions. The possible formation of side-phase non-amorphous products, such as GdF_3_, BaF_2_, or non-reacted metal chlorides, has been excluded based on full profile analysis.

Table 3 depicts the expected (i.e., estimated in accordance with the initial precursor’s loading) and actual Tb ions content for each synthesized sample, while Table S1 extends elemental analysis results to the entire composition of the synthesized samples. One observes a monotonic increase in the terbium content, while the actual number of the observed Tb^3+^ ions is in close correspondence with the number of Tb^3+^ precursors loaded into the synthesis. An actual Gd/Tb ratio also exhibit values close to the theoretical ones. At the same time, the intercalation of Tb^3+^ ions in the smallest amounts (i.e., 5Tb sample) into the BaGdF_5_ lattice has significantly improved in comparison with our previous work [35]. Overall, we found that MF synthesis resulted in slightly more accurate doping, yielding a lower difference between the expected and the actual Tb-contents compared with previously reported MW synthesis (with the only exception of 10Tb sample) [35]. This better Tb^3+^:Gd^3+^ substitution efficiency was likely achieved due to better miscibility of components in microchannels compared to mixing in a large volume of reagents used in MW synthesis.

**Figure 3:**
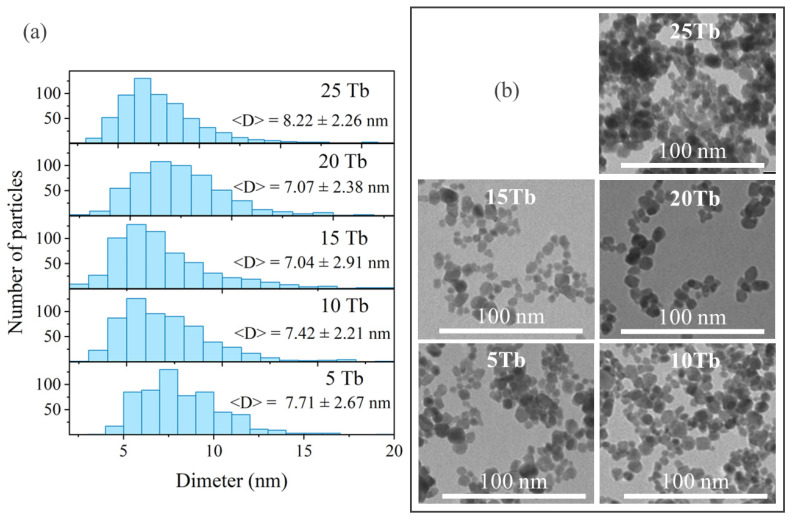
BaGd_1−x_F_5_:Tb_x_ samples, obtained by MF synthesis: (**a**) particle size distribution according to the TEM; (**b**) TEM images.

Analysis of TEM images (see Figure 3) showed the formation of spherically and irregularly shaped nanoparticles with an average particle size of 7–8 nm. In contrast to classical microwave synthesis (see Figure S5), the addition of water to EG does not lead to an increase in the size of nanoparticles but even slightly reduces it while maintaining monodispersity (see Figure S6). As can be seen from the DLS data measured for the 15Tb sample (Figure S7), the average hydrodynamic size of nanoparticles in the colloidal solution obtained by the MF method was around 22 nm, while the size distribution obtained for MW was shifted toward bigger particle size, yielding an averaged hydrodynamic size of around 34 nm. It is worth noting that the nanoparticle hydrodynamic size is always greater than their actual size (typically accessed by TEM), because it considers the adsorption and diffusion layers of solvent molecules on the particles’ surface. In this particular case, larger values obtained for the hydrodynamic size of the NPs might also be related to small but not negligible amounts of EG molecules attached to the particle surface. Indeed, the traces of EG were observed by IR-spectroscopy in ATR mode even when the number of washing cycles was increased up to eight times (Figure S8).

**Figure 4:**
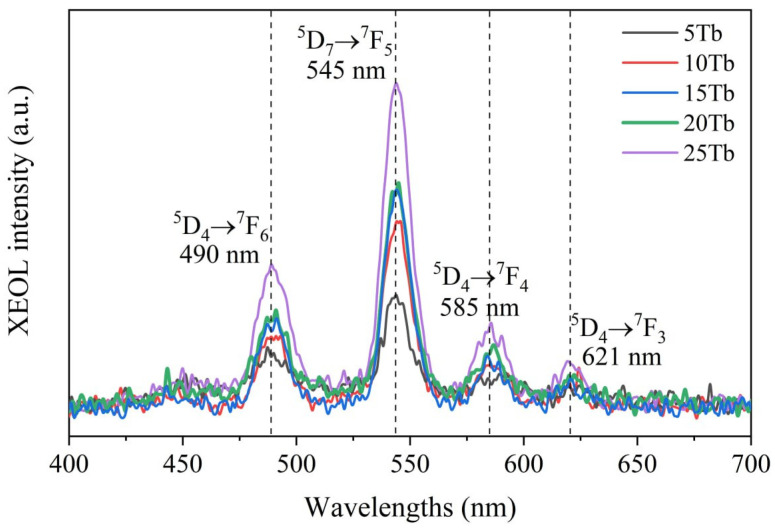
X-ray-excited optical luminescence (U = 35 kV, I = 3.0 mA) spectra of BaGd_1−x_F_5_:Tb_x_^3+^ measured in situ.

Finally, Figure 4 demonstrates the XEOL spectra measured in situ for the synthesis with different flow rate ratios for GdCl_3_ and TbCl_3_ reagents solutions (see Table 2), which allowed obtaining of the samples with varied actual content of Tb^3+^, as revealed by XRF composition analysis (see Table 3). Each of the curves reported in Figure 4 was obtained by averaging over six spectra collected in flow mode (ca. 40 s per spectrum) and then scaled in accordance with the NPs concentration in the collected synthesis products. Moreover, to make sure that XEOL spectra were measured for particular synthesis conditions after each combination of reagent flows, the system was flushed with a solution of 75EG in deionized water. Figure S9 demonstrates the efficiency of such a “cleaning step” by reporting XEOL spectra collected on the empty XEOL chip before synthesis and after the cleaning stage.

The obtained X-ray-excited luminescence spectra have a typical profile for Tb^3+^ doped systems and demonstrate characteristic radioluminescence (RL) peaks of Tb^3+^ ions at 490 nm, 545 nm, 585 nm, and 620 nm associated with electronic transitions from the lower excited ^5^D_4_ state to the ground states ^7^F_J=3,4,5,6_, respectively, with the dominant emission line at 545 nm (green), which correspond to magnetic dipole transition with ΔJ = 1. Despite rather different actual Tb contents (see Table 2), the intensity of emission at 545 nm for 10Tb and 15Tb samples was found to be nearly the same, which might be related to slightly different fluorescence quenching in these samples or some systematic uncertainty in concentrations of the NPs in MF synthesis product. The most intense XEOL signal was obtained for the 25Tb sample with an actual Tb content equal to 3.63 at.% (which corresponds to our previously reported results obtained for the series of Tb-doped synthesis by means of the MW procedure [35]).

Finally, to compare nanophosphors synthesized by MW and MF, photoluminescent spectra were recorded upon direct excitation of ^8^S_7/2—_^6^I_J_ adsorption band (λ_ex_ = 274 nm) of Gd^3+^ using the third harmonic of LOTIS TII tunable laser LT-2211A (τ = 10 ns, ν = 10 Hz). The collected spectra are reported in Figure S10 and confirm the efficient Gd^3+^ → Tb^3+^ energy transfer for both samples, exhibiting very weak intensity of Gd^3+^ ions (clearly not visible in XEOL spectra) compared to the main emission lines of Tb^3+^ ions.

To summarize, adapting BaGdF_5_-based nanophosphor synthesis in MF mode can solve several problems of classical syntheses. First, due to the small volumes of reaction mixture present in microfluidic synthesis fast heat convection provided, reaction temperature might be decreased down to 100 °C while synthesis time can be notably decreased from 12–24 h (in ST batch synthesis) and 1–2 h (in MW approach) to 6 min, retaining the structure and crystallinity similar to those obtained in MW synthesis (See Figure S2 and Figure S12). In addition, the ability to vary flow rates in “on the fly” mode allow for the performance of many syntheses with different percentages of doping elements in a short time and with reduced (compared to the classical synthesis) consumption of the reagents. Second, due to the good mixing of precursors in small volumes, an increase in the terbium intercalation efficiency is observed, which might be particularly important if one needs to obtain samples with a low percentage of doping elements. Finally, the adaptation of the synthesis reaction in microfluidic mode preserves such important properties as single-phase products and monodispersity of the synthesized particles in solutions.

### 2.3. Single-Stage Microfluidic Synthesis of BaGdF_5_:Tb@RoseBengal Nanocomposite

To date, only a few examples of Ln-doped nanocomposites obtained on the microfluidic platform have been reported in the literature. J. Ma and co-workers have reported the synthesis of Ce/Eu/Tb-doped KGdF_4_ matrix within a microfluidic reactor [31], which was further functionalized by hyaluronic acid in the microfluidic reactor as a separate stage of the synthesis post-treatment. M. Iskawa and E. Guidelli very recently reported a microfluidic synthesis of the nanocomposite consisting of Eu-doped scintillating NPs and layered structure of polyacrylic acid (PAA) and Methylene Blue as a photosensitizer [41]. However, while the synthesis of the GdF_3_:Eu was performed using a microfluidic reactor with varied temperatures and synthesis reaction (from 30 s to 10 min), the subsequent NPs surface modification was performed as a post-synthetic multi-stage procedure involving several long-last centrifugation and impregnation steps.

**Figure 5:**
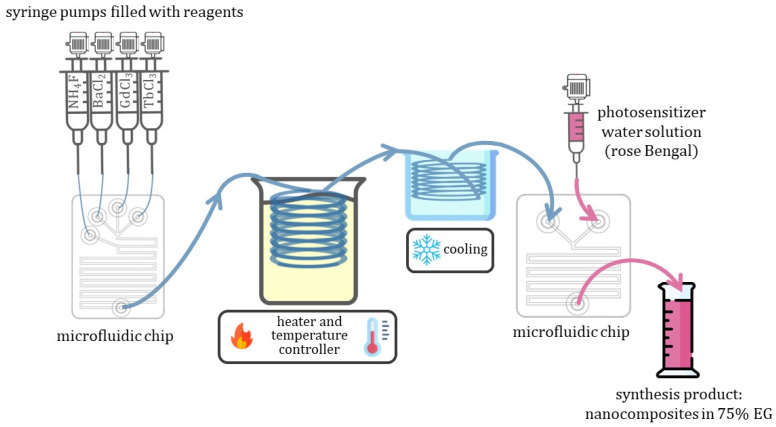
Scheme of BaGdF_5_:Tb@RB nanocomposite single-stage microfluidic synthesis.

At the next stage, we consider the possibility of a simple single-stage flow-through synthesis protocol for obtaining nanoconjugates of BaGdF_5_:Tb^3+^ as an X-ray-excited nanophosphor and rose bengal (RB) as a suitable photosensitizer with sufficient overlap in the emission spectrum of scintillating NPs and absorption band of RB. The scheme of microfluidic synthesis setup for obtaining a nanocomposite in a single-stage procedure is shown in Figure 5. In nanocomposite synthesis, 0.1 M chloride solutions and 0.55 M ammonium fluoride solution were used.

After the reaction mixture sequentially passed the first four-inlet microfluidic mixer, silicon oil bath, and colling water-filled bath, it was further mixed with a constant flow of rose bengal aqueous solution (0.0625 mg/mL or ca. 6.4⸱10^−5^ M) supplied to the system through a second microfluidic two-inlet reactor of meander topology with a flow rate of 30.84 μL/s (equal to the total flow rate of all four reagents supplied). Then, the obtained pinkish colloidal solution was collected to the glass vessel and centrifuged at 15,000 rmp for 5 min in order to separate weakly bounded PS molecules, while the supernatant was analyzed by measuring its optical density by UV-vis spectroscopy. In order to obtain nanocomposites, the collected reaction mixture was centrifuged at 15,000 rmp and thoroughly washed with DI water three times. Again, some of the samples were dried at 60 °C and collected in the form of pink-colored powder, while the rest were re-dissolved in water and form a stable colloidal solution.

**Figure 6:**
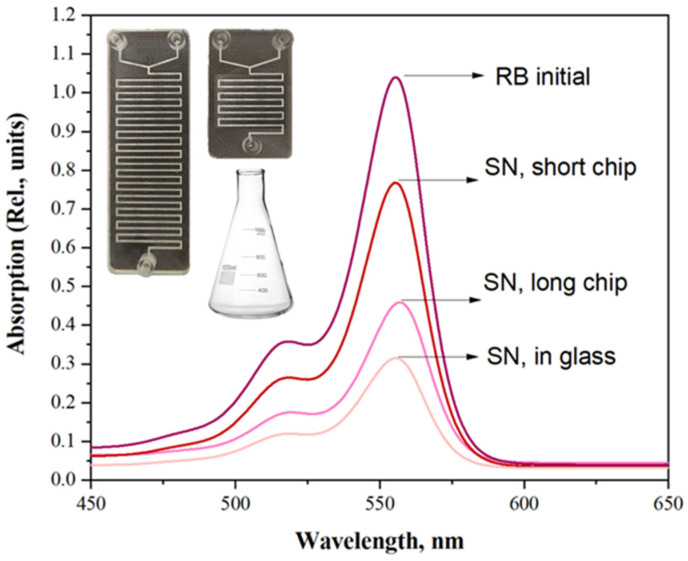
UV-vis spectra collected for initial rose bengal aqueous solution used for NPs impregnation as well as UV-vis spectra collected for supernatants (SN) obtained from impregnation in single-stage microfluidic procedure using short and long MF chips, as well as obtained from the conventional impregnation technique (“in glass”) after 30 min of active magnetic steering.

To estimate the effect of chip length on impregnation efficiency, two types of two-components (two-inlets) mixing chips were used: short (total length of the channel around 16 cm) and long (total lengths of the channel around 47 cm); see insets in Figure 6. Nanocomposite syntheses were performed in accordance with a scheme reported in Figure 5.

At the same time, the classic method of impregnation “in glass” was implemented to compare the efficiency of the NPs@RoseBengal conjugation in a microfluidic setup. Previously obtained by microfluidic synthesis, unwashed NPs and 75EG solution were added to the equal volume of an aqueous RB solution in a glass flask and mixed intensively by a magnetic stirrer for 30 min at room temperature. The results of impregnation were centrifuged at 15,000 rmp for 5 min, and the optical absorption spectrum of the supernatant was collected (Figure 6).

The obtained results demonstrate that for a short MF chip, a relatively low impregnation level was obtained (up to 75% of RB molecules remain unbounded to the NPs). While utilization of a longer chip allows for a significant improvement in the impregnation efficiency, decreasing the number of non-interacting RB species down to 45%, being still slightly less efficient compared with the classic “in glass” approach (only 30% of remaining unbounded RB molecules) but remaining in the concept of single-stage process of nanocomposite synthesis.

### 2.4. Cytotoxicity and Flow Cytometry Results

**Figure 7:**
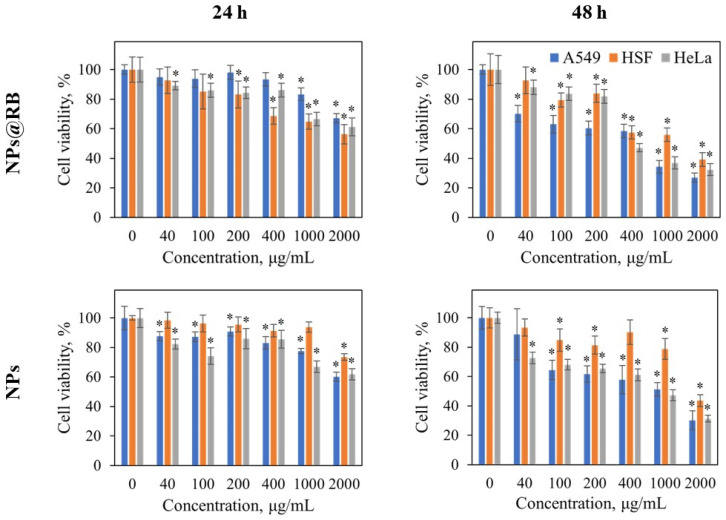
Viability of A459, HSF, and HeLa cells treated with MF-synthesized nanoparticles BaGd_0.85_F_5_:Tb_0.15_ (NPs) and nanocomposites BaGd_0.85_F_5_:Tb_0.15_ @RoseBengal (NPs@RB) after incubation for 24 h and 48 h. Cell viability in the variant without treatment was taken as 100%. *—*p* ≤ 0.05 in comparison with untreated variant. Data represented as average % of viability ± SD.

Cytotoxic effects of NPs were estimated using MTT-assay. Both nanoparticle types investigated reduced the viability of A459, HSF, and HeLa cells in a concentration- and treatment-time-dependent manner (see Figure 7). Nevertheless, the cytotoxic effects of the nanomaterials were moderate and reached significant values only when treatment time of 48 h and high concentrations (400 µg/mL–2000 µg/mL) were combined.

Detection of nanoparticles in cells by flow cytometry is one of the methods to assess the capture and retention of nano-objects by cells [42]. Depending on the optical properties of the nanoparticles, the increase in the side scattering signal (SSC) for the cells might be modified compared with the control line. Indeed, uptaken nanoparticles or composites being localized inside cells and/or on the cell membrane increase the cell optical inhomogeneity that is reflected in the increase in the SSC signal [43]. In this study, we evaluated the SSC changes in only live cells defined as PI-negative. Since we wished to demonstrate the fundamental possibility of the synthesized nanoparticle uptake by cells, treatment with agents at a concentration of 400 μg/mL for 12 h was chosen for flow cytometry, which kept most of the cells viable (see Figure S11).

A significant change in the SSC signal was revealed in treated cells in comparison to the control (Table 4). For only one variant (HeLa, NPs@Rb), we recorded no statistically significant increase in SSC after incubation. The effect of agents was most pronounced in HSF cells, for which a 1.63- and 1.88-fold increase in SSC was demonstrated after treatment with NPs@Rb and NPs only, respectively. The data obtained allowed us to validate the assumption about the ability of cells to capture synthesized nano-objects. The higher level of SSC signal obtained for HSF declares that investigated nanoparticles and nanocomposites are absorbed by this line more readily.

### 2.5. Long-Term Monitoring of Nanocomposite Biodistribution via Micro-CT

**Figure 8:**
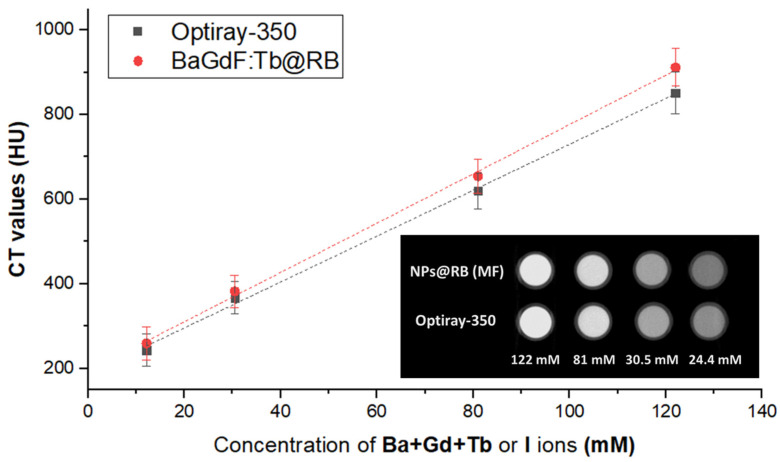
Comparison of X-ray attenuation for MF-synthesized BaGdF_5_-nanocomposites and commercial iodine-based contrast Optiray-350^®^ at different molar concentrations. Molar concentrations estimated considering heavy elements only.

Prior to the in vivo CT imaging experiments, an X-ray attenuation capability of the synthesized nanocomposite was estimated in comparison with commercial nonionic iodine-based contrast agent Opriray-350^®^. For this purpose, an aqueous solution of BaGd0._85_F_5_:Tb_0.15_ conjugates obtained in MF with impregnation in a long chip was com-pared with Optiray-350^®^ diluted with deionized water in order to have similar molar concentrations of heavy ions in synthesized sample (i.e., Ba + Gd + Tb) and I ions in commercial contrast. In accordance with previous reports on the X-ray attenuation efficiency of BaGdF_5_ matrix [32,34,44], the synthesized nanocomposites slightly outperform the contrast capability of commercial iodine-based contrast Optiray-350^®^ (See Figure 8), likely due to the fact that Ba and Gd more efficiently absorb high-energy X-ray photons because of larger X-ray attenuation coefficient compared with I ions (at 60 kV, Ba = 8.51, Gd = 11.75 and I = 7.58 cm^2^ g^−1^ [45]).

**Figure 9:**
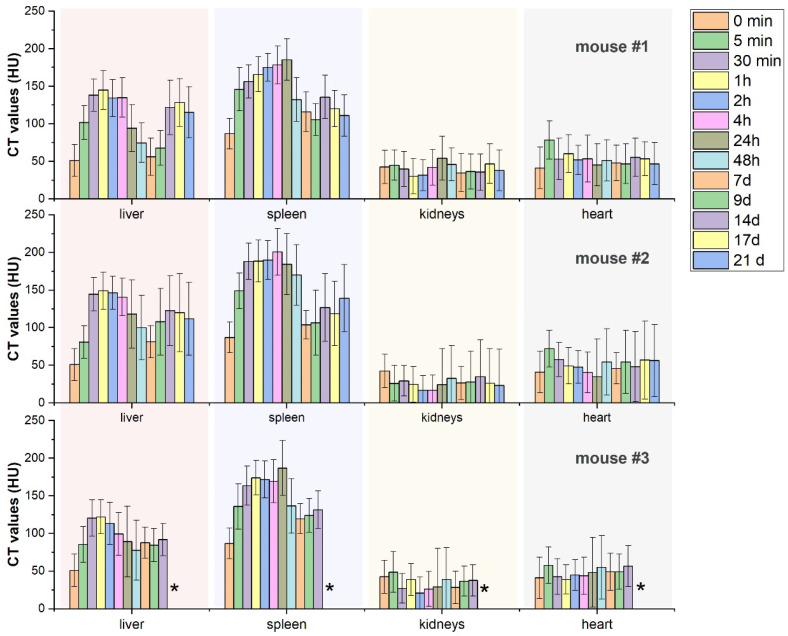
Results of quantitative analysis of MF-synthesized nanocomposite biodistribution in liver, spleen, kidneys, and heart accessed at different time intervals after tail vein injection of nanocomposites (the results reported for each mouse individually, mouse #3 was withdrawn from experiment after 2 weeks of observation, and * symbol corresponds to the skipped columns). Data reported as “0 min” scan correspond to the CT values estimated for intact mouse.

**Figure 10:**
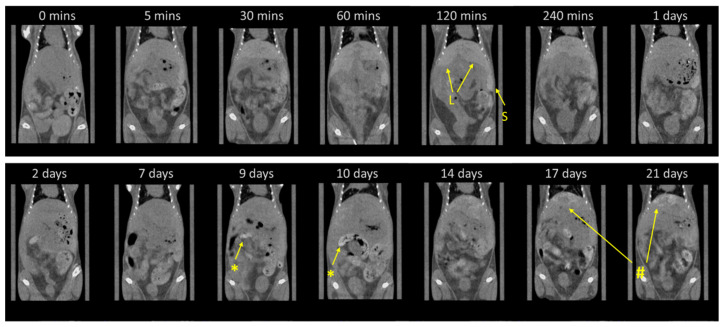
Visualization of the abdominal cavity for mouse #1 (as reported in Figure 9) assessed by X-ray micro-CT for intact mouse (0 min scan) and at different time intervals after tail vein injection of nanocomposites. L and S depict the localization of the liver and spleen, respectively, where the accumulation of injected nanocomposites was predominantly observed. *—The high contrast area in the colon due to the presence of mineralized mice’s feed; #—Uneven distribution of the composite in the median lobe of liver tissue formed in the third week of observation.

At the next stage, the biodistribution of the synthesized nanocomposites was assessed via in vivo micro-CT tomography. For this experiment, MF-synthesized BaGd_0.85_F_5_:Tb_0_._15_@RB composites obtained by impregnation procedure were thoroughly (eight times) washed with deionized water and subsequently obtained aqueous solution (concentration ca. 35 mg/mL) was injected in the tail vein of three BALB/c mice. Despite the relatively high concentration of heavy ions (ca. 0.77 mg per 1 g of mice weight) chosen for reliable contrast level for quantitative estimation, no acute toxicological signs were observed during the entire period of observations—3 weeks. The results of the quantitative assessment are reported in Figure 9, while the visualization of the abdominal cavity for mouse #1 at different intervals after injection is reported in Figure 10. In agreement with earlier reports in the literature on the biodistribution of PEG-modified BaGdF_5_ nanoparticles [44], during the first day, nanocomposites predominantly accumulated in the liver and spleen. It is worth noting that for the liver, the maximum contrast was observed around 1 h after injection, while for the spleen, the maximum concentration was observed in the period from 4 h to 24 h.

For all mice used in the experiment, a small but systematic increase in contrast of heart was observed right after the nanocomposite injection (5 min), revealing the presence of the agents in the blood system of mice at relatively high concentrations. No changes or stochastic changes in the contrast ability of kidneys have been observed during the entire period of observation, declaring no accumulation of the nanocomposites in detectable amounts. An unusual behavior has been obtained for long-term observation of nanocomposite accumulation in the liver and spleen. In the first case, as soon as after 24 h, a systematic decrease in the concentration was observed, while for spleen contrast, a sharp decline for two of the three mice was observed on the 2nd day. The lowest concentrations of nanocomposites for the liver and spleen were observed for the period of 7–9 days after injection, and CT values obtained for the tissues in both cases were only 15–20% higher than for the intact mice (0 min scan). Surprisingly, the significant reduction in the NP concentrations was subsequently changed by re-accumulation predominantly in the liver (clearly observed for mouse #1 and #2 on the timescale of 2nd and 3rd week of observation). The noticeable increase in the contrast of the spleen was also detected for the 14th, 17th, and 21st days of observations. Moreover, upon undesirable re-accumulation, an uneven character of nanoagent distribution in the median lobe of the liver was observed starting from 14 days after injection and more clearly observed for the 17th and 21st days of observations (See Figure 10), while no inhomogeneous region upon re-accumulation was detected for spleen.

Due to ethical reasons, an in vivo experiment was stopped after 21 days for mice #1 and #2, while mouse #3 was withdrawn from the experiment after 14 days when the effect of uneven re-accumulation had not yet been sharply observed. The tissues of the spleen, liver, kidney, and lungs were collected for further investigations posthumously.

In summary, the predominant accumulation of the nanocomposites was observed in the liver and spleen as organs responsible for blood filtration characterized by a well-developed vascular system. For both organs, the essential decrease in NP accumulations was observed during the first week, accompanied by subsequent re-accumulation for the second and third weeks. We propose that in the period from the 2nd to the 9th days, the NPs were gradually released back into the blood system, thus declaring that at least a fraction of the supplied nanoagents does not experience phagocytosis by the liver and spleen cells. This hypothesis, however, should be accurately verified which will be the subject of our further work. The sensitivity of the micro-CT method is not sufficient to detect moderate and smooth variation in the composite concentrations in the blood system (indeed, as demonstrated in Figure 9, the systematic increase in CT-value of the heart region was detected only for a 5 min scan right after injection when the increase in NP concentration was essential).

Clearly, an uneven character of nanocomposite re-accumulation in the liver can be considered as an undesirable negative effect, while the mechanism of this process remains unclear and will be the subject of our further investigations. We should also mention that no data on the peculiarities of long-term biocirculation of BaGdF_5_-based (or other lanthanide fluorides) nanoparticles or nanocomposites have been reported in the literature so far.

## 3. Materials and Methods

### 3.1. Microfluidic Setup and Microreactors

The synthesis discussed in this work was performed using a homemade microfluidic setup (MF-1) based on electric engines of NEMA-23 stepper motors, syringes (Runze New Technology Development Co., Nanjing City, Jiangsu Province, China) that are made of chemically resistant materials (PTFE, borosilicate glass and stainless steel), as well as dedicated software which allowed us to control flow rate and dosage for each syringe pump individually. Microfluidic chips were printed using the Asiga UV MAX 3D printer (Asiga Co., Sydney, Australia) using the DLP (digital light processing) technique. Syringes with a volume of 10 mL were filled with precursor solutions. Silicone tubes were used to connect MF synthesis system elements to each other. A PTFE tube 1.5 m long and with an internal diameter of 2.5 mm was used in a heating oil bath filled with silicone oil. An IKA C-MAG HS 7 heating furnace with a magnetic stirrer was used to heat up and keep an oil bath at a constant temperature.

The MF chips were equipped with a passive micromixer of conventional meander topology for mixing two- and four-components with a channel width of 500 μm (see Figure 1 and Figure 5).

### 3.2. X-ray-Excited Optical Luminescence

XEOL signal was recorded by using a homemade experimental setup based on an X-ray tube with a Cu target as an X-ray source and an Agilent Cary Eclipse fluorescence spectrophotometer to detect X-ray-excited luminescence signal, as described elsewhere [46]. The emission slit of the fluorescence spectrometer was set to 10 nm, and the X-ray tube was operating at *U* = 35 kV and *I* = 3.0 mA.

To measure XEOL spectra, a special MF chip (see inset at Figure S3) equipped with a flat rhombic-shaped reservoir 2 × 2 cm and total volume around (600 μL) were utilized for two reasons: (i) to maximize the area from where the signal can reach a fluorescence detector (the sample position was not aligned perfectly at the focal point of fluorescence detector optical scheme); (ii) to reduce the linear speed of the synthesis mixture at the area of X-ray irradiation and XEOL signal acquisition. The reservoir of the chip was printed without a top wall, which subsequently was covered with a thin glass window and tightly glued with FunToDo photopolymer resin to make the cell more optically transparent and prevent loss in the luminescence signal.

### 3.3. Other Characterization Techniques

X-ray powder diffraction (XRD) was measured by means of Bruker D2 PHASER (Bruker AXS Inc., Fitchburg, WI, USA) using Cu K radiation (λ = 1.5406 Å) at 30 kV, 10 mA, and the following parameters: *2θ* range from 5 to 90°; step size of 0.01°. FEI Tecnai G2 Spirit BioTWIN (FEI, Hillsboro, OR, USA with accelerating voltage of 80 kV) was used for TEM visualization of the synthesized samples. The elemental composition was analyzed using micro-focused X-ray fluorescence spectrometer M4 TORNADO (Bruker, Billerica, MA, USA). The data were collected at 20–25 spots (each 25 × 25 μm) of a sample surface, 30 s acquisition for each spot. Hydrodynamic particle size distribution data were measured using a NANO-flex particle size analyzer (MicroTrac, GmbH, Haan, Germany). The data were accumulated over five consecutive measurements of 2 min each and then were summed considering the dynamic viscosity of the solution. The signal collected for a pure solvent (water) was subtracted from the background. The UV–vis spectra of photosensitizers and supernatant obtained after NP impregnation were collected using a Shimadzu UV-2600 double-beam spectrophotometer (Shimadzu Co., Kyoto, Japan).

### 3.4. Cytotoxicity Test

Human lung adenocarcinoma A549, cervical adenocarcinoma HeLa cells (both lines from Russian Collection of Cell Cultures of Vertebrates (CCCV)) and human skin fibroblasts HSF previously obtained from a healthy donor were cultured in DMEM (Gibco, Thermo Fisher Scientific, Waltham, MA, USA) supplemented with 10% FBS (BioSera, Cholet, France), 2 mM/L-glutamine, 100 units/mL of penicillin, and 100 μg/mL of streptomycin.

MTT assay was used to determine the changes in the viability of A549, Hela, and HSF cells under the nanoparticles’ action. Briefly, cells were seeded in 96-well plates with a density of 1 × 10^4^ cells per well and left overnight for the attachment at 37 °C and 5% CO_2_ in a humidified atmosphere. Then, the medium in wells was replaced with a fresh one containing various NP concentrations (40 μg/mL–2000 μg/mL), and plates were incubated for 24 h and 48 h. After incubation, the medium in the wells was changed to MTT (0.5 mg/mL final concentration) containing medium for 3 h. Then, the medium was aspirated, and 100 µL of dimethyl sulfoxide per well was added to dissolve the formazan crystals. The optical density of the formazan solution in the wells was measured using a BioRad xMarkTM Microplate Spectrophotometer (Bio-Rad, Hercules, CA, USA) at a wavelength of 570 nm.

### 3.5. Flow Cytometry

A BD FACSCanto II (BD Biosciences, USA) flow cytometer equipped with a 488 nm laser was used in this study. For flow cytometry, cells were plated in T25 culture flasks (SPL Life Sciences, South Korea) (5 × 10^4^ cells/mL) for 24 h; then, the medium was replaced with a fresh one containing 400 μg/mL of NPs for 12 h. After incubation, cell monolayers were washed twice with 0.01 M phosphate-buffered saline (PBS, pH 7.2) to remove nanoparticles that had not bound to the cells and were then trypsinized, centrifuged, and suspended in 1 mL of PBS. To determine viability in cytometrical studies, cell cultures were stained with 5 μg/mL of propidium iodide (PI), which stained cells with compromised membrane integrity, for 2 min at room temperature and then stored on ice before analysis. At least 10,000 events per sample were analyzed. The data were analyzed with BD FACSDiva Software https://www.bdbiosciences.com/en-us/products/software/instrument-software/bd-facsdiva-software (accessed on 2 November 2023). Only viable cell populations were taken for the analysis.

### 3.6. Statistical Analysis

All experiments with cell cultures were performed with at least three repeats in each run. For all tests, significant differences were reported at *p* < 0.05 using the nonparametric Mann–Whitney U-test for the results of the MTT assay and the Two-Sample Z-Test for flow cytometry data.

### 3.7. Micro-CT In Vivo Diagnostics

For the in vivo experiments on biodistribution of the nanocomposites, intact BALB/c male mice (three mice were used in the experiment, ca. 3 months old, 34–35 g weight) were anesthetized with 2% isoflurane (Laboratories Kari-zoo, S.A., Barcelona, Spain) using a dedicated RAS-4 anesthesia device (Perkin Elmer, Boston, MA, USA). An aqueous solution of BaGd_0.85_F_5_:Tb_0.15_ sample (170–200 µL, the concentration of ca. 35 mg/mL; 0.2 mg of NPs per 1 g of animal weight) was administrated in a tail vein. At the different time intervals after nanocomposite injection, micro-CT images were taken with the following parameters: tube voltage = 80 kV; tube current = 90 µA; FOV was restricted to an 86 × 72 mm rectangle area; voxel size = 140 µm. For each CT scan, acquisition time was equal to 4 min, corresponding to the total radiation dose of 136 mGy. All the animal experiments were carried out in accordance with the Guide for the Care and Use of Laboratory Animals and accepted by the local ethics committee [47].

## 4. Conclusions

In this work, we demonstrated that the synthesis of BaGdF_5_:Tb^3+^ can be successfully adopted in a microfluidic flow-through regime at temperatures and thermal treatment times as low as 100 °C and 6 min, respectively. Moreover, it was shown that the significant decrease in the synthesis time regulated by the total flow of the reagents did not lead to the loose of sample crystallinity. TEM imaging declares the formation of nanoparticles with spherical and irregular shapes with an averaged diameter of 7–9 nm. It was shown that in microfluidic synthesis, the number of doping elements could be efficiently controlled by the variation of Gd^3+^ and Tb^3+^ precursors flow rates, while the target property of synthesized samples—luminescence under X-ray excitation (XEOL intensity)—could be monitored in situ during the synthesis. The photoluminescence collected upon the direct excitation of Gd^3+^ ions at λ_ex_ = 274 nm by laser source revealed an efficient Gd^3+^ → Tb^3+^ ions energy transfer. In addition, it was shown that BaGdF_5_:Tb@RoseBengal nanocomposites could be obtained in a simple single-stage procedure by mixing in additional microreactor the synthesis product containing NPs in EG solution and aqueous solution of rose bengal as photosensitizer. Both bare NPs and their conjugates with PS demonstrate moderate cytotoxicity, while flow cytometry data indirectly confirm efficient cellular uptake of the samples by human lung adenocarcinoma A549 and human skin fibroblasts HSF and less evidently by HeLa cells. Also, the in vivo toxicity test on balb/c mice does not demonstrate any acute toxicological signs, while the biodistribution of the samples assessed by X-ray micro-CT reveals the unusual character of NP accumulation consisting of a gradual and substantial decrease in the NPs concentration in the liver and spleen after the first week of observation, with their subsequent uneven re-accumulation in the median lobe of the liver.

## Figures and Tables

**Table 1:** Summary of the different test of reaction conditions in MF synthesis.

**Solvent Composition**	
X% of EG	Particle concentration, mg/mL
100 EG	1.5–2
75 EG	10–12
50 EG	chip clogging
25 EG	chip clogging
0 EG	chip clogging
**Synthesis time (total flow rates)**	
Time	Particle concentration, mg/mL
2 h	chip clogging
1 h	10–12
30 min	10–12
12 min	10–12
6 min	10–12
Metals chloride (precursors) concentration *	
Concentration, mol/L	Particle concentration, mg/mL
0.1	10–12
0.2	17–19
0.3	25–30
0.4	chip clogging
0.5	chip clogging

* Concentrations of fluoride precursor (NH_4_F) were modified, respectively, keeping proportion 5F ions per 1 Gd/Tb/Ba ion.

**Table 2:** Precursor flow rates used in MF synthesis to obtain different levels of doping element concentrations. Sample naming hereinafter correspond to the expected substitution of Gd by Tb ions (e.g., 5Tb corresponds to expected level of 5% Gd-Tb substitution).

Sample	Precursor Flow Rates, μL/s			
	Ba^2+^	Gd^3+^	Tb^3+^	F^−^
5Tb	10.28	9.77	0.51	10.28
10Tb		9.25	1.03	
15Tb		8.74	1.54	
20Tb		8.22	2.06	
25Tb		7.71	2.57	

**Table 3:** Cell parameters of the BaGd_1−x_F_5_:Tb_x_^3+^ samples calculated from the full-profile XRD analysis and actual Tb content retrieved from XRF, compared with expected one.

Sample	Expected Tb Content, at.%	Actual Tb Content, at.%	Cell Parameters, Å	Cell Volume, Å^3^	Goodness of Fit (GOF)	R-Factor
5Tb	0.71	0.66	5.9388 (14)	209.46 (8)	1.06	0.1506
10Tb	1.43	1.29	5.9408 (14)	209.67 (9)	1.06	0.1449
15Tb	2.13	2.11	5.9355 (12)	209.11 (7)	1.03	0.1275
20Tb	2.86	2.86	5.9352 (16)	209.08 (10)	1.08	0.18
25Tb	3.57	3.63	5.9337 (11)	208.92 (7)	1.01	0.1217

**Table 4:** Side scatter signals of A549, HSF, and HeLa cells after 12 h incubation with 400 µg/mL of NPs and NPs@RB composites.

Sample	A549	HSF	HeLa
Control	28.156 ± 8.823	32.261 ± 12.467	31.060 ± 10.525
NPs@RB	40.398 ± 13.794 *	52.504 ± 31.848 *	32.343 ± 19.897
NPs	45.946 ± 13.134 *	60.513 ± 30.340 *	37.061 ± 15.484 *

*—*p* ≤ 0.05 in comparison with untreated variant (control). Data represented in r.u. as mean ± SD.

## Data Availability

The database and the indicators analyzed in the work are held by the authors Zaira Gadzhimagomedova (zgad@sfedu.ru), Ilia Pankin (pankin@sfedu.ru), and Vladimir Polyakov (vlpolyakov@sfedu.ru).

## Supplementary Materials

The following supporting information can be downloaded at https://www.mdpi.com/article/10.3390/ijms242417159/s1.
