# Supplementary material for: Single-Stage Microfluidic Synthesis Route for BaGdF5:Tb3+-Based Nanocomposite Materials: Synthesis, Characterization and Biodistribution
Source: Int J Mol Sci. 2023 Dec 5;24(24):17159. doi: 10.3390/ijms242417159 (PMC10742823; doi:10.3390/ijms242417159)
Supplement: Supplementary file 1 [file ijms-24-17159-s001.zip › ijms-2727818-supplementary.pdf]

Supporting information

Single-Stage Microfluidic Synthesis Route for $BaGdF_5:Tb^{3+}$-Based Nanocomposite Materials: Synthesis, Characterization and Biodistribution

Zaira Gadzhimagomedova [1], Ilia Pankin [1,*], Vladimir Polyakov [1,*], Darya Khodakova [2], Pavel Medvedev [1], Pavel Zelenikhin [3], Nail Shamsutdinov [3], Sergey Chapek [1], Anna Goncharova [2] and Alexander Soldatov [1]

[1] The Smart Materials Research Institute, Southern Federal University, Rostov-on-Don 344090, Russia; zgad@sfedu.ru (Z.G.); pmedvedev@sfedu.ru (P.M.); chapek@sfedu.ru (S.C.); soldatov@sfedu.ru (A.S.)
[2] National Medical Research Centre for Oncology, Rostov-on-Don 344037, Russia; hodakovadv@rnioi.ru (D.K.); goncharovaas@rnioi.ru (A.G.)
[3] Institute of Fundamental Medicine and Biology, Kazan Federal University, Kazan 420008, Russia; pavel.zelenikhin@kpfu.ru (P.Z.); michaeldermoon@gmail.com (N.S.)
* Correspondence: pankin@sfedu.ru (I.P.); vlpolyakov@sfedu.ru (V.P.)

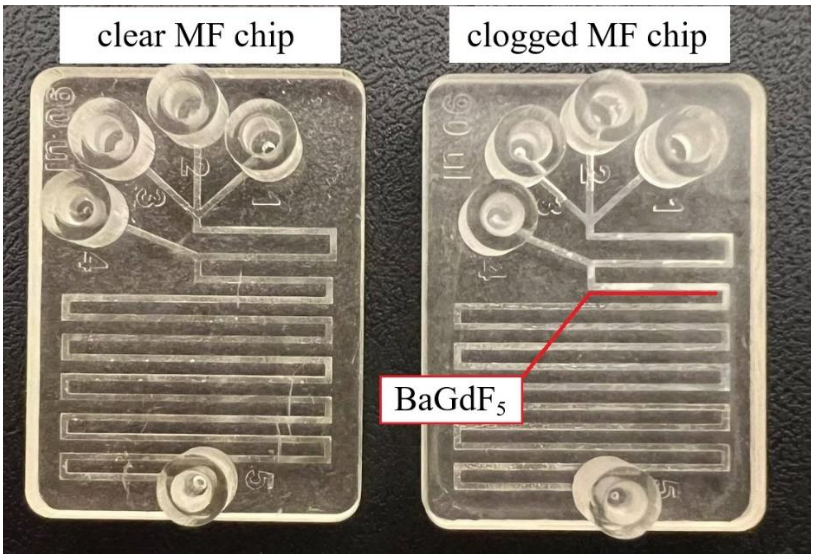

Figure S1. Image of a clean MF chip (left) and a MF chip clogged with $BaGdF_5$ (right)

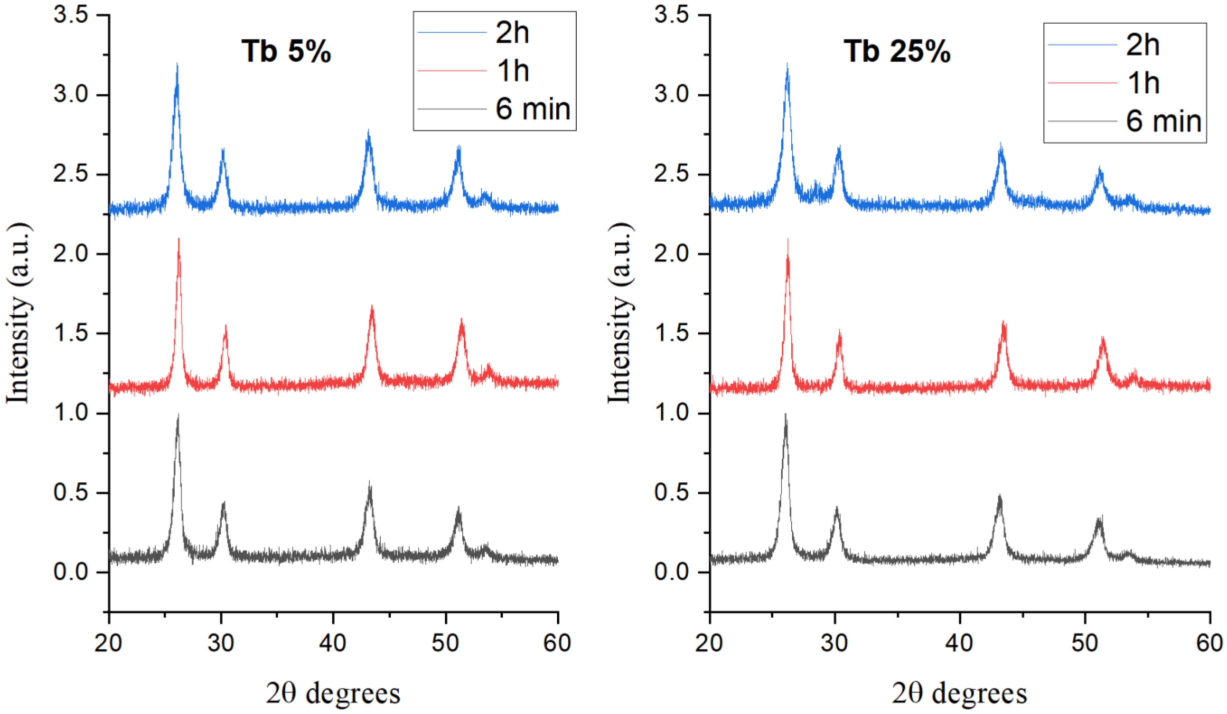

Figure S2. X-ray diffraction patterns for the samples with theoretical 5% and 25% of Tb-Gd substitutions obtained within microfluidic with different synthesis time 2h (total flow rate ca. 1.54 μL/s), 1h (ca. 3.08 μL/s) and 6 min (30.84 μL/s). XPRD declare no notable loss of crystallinity upon significant reduction of the synthesis time.

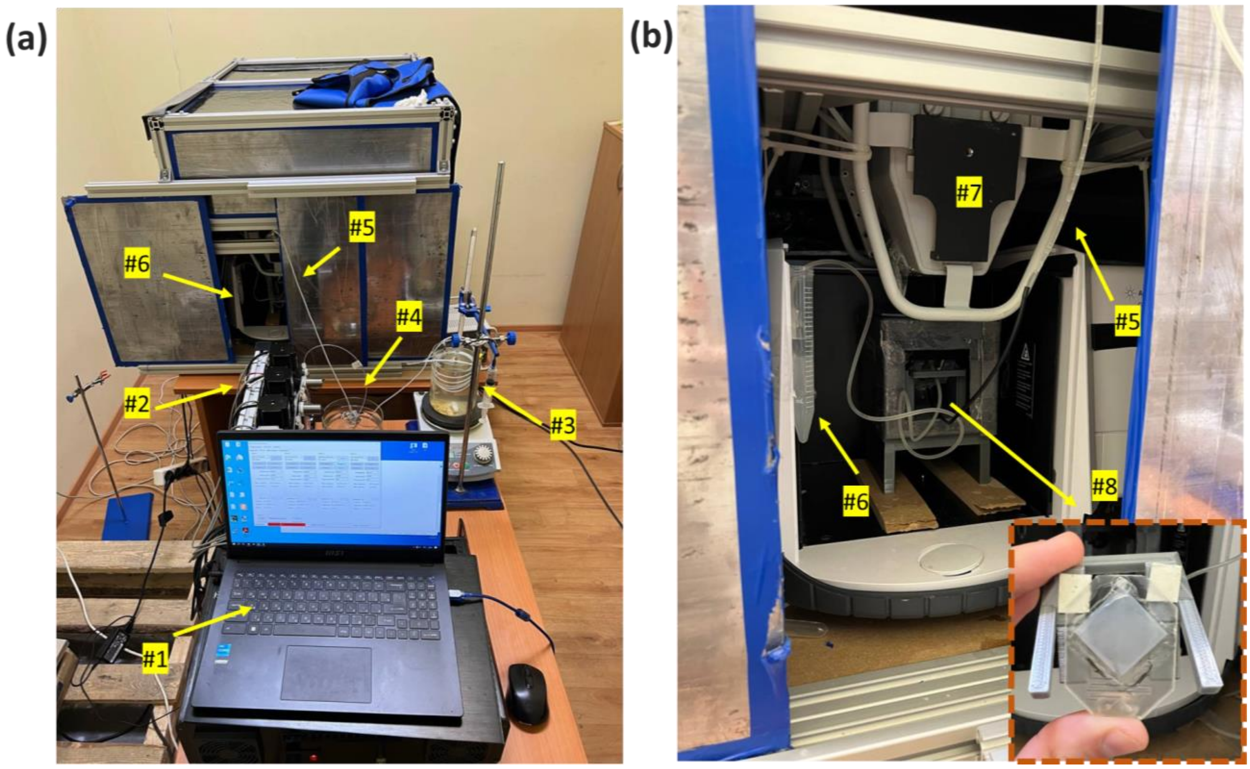

Figure S3. Photo of microfluidic setup used for the synthesis of $BaGd_{1-x}F_5:Tb_x$ samples with *in situ* XEOL acquisition: **(a)** an overall view; **(b)** sample environment view. In detail: **(a)** #1 – laptop with the software which control the system of syringe pumps (flow rates and volumes); #2 – syringe pumps; #3 – oil bath kept at T = 100 °C with an immersed PTFE tube of 1.5 m length; #4 – colling bath filled with water and kept at RT; #5 – silicone pipe entering the lead cover of the setup for XEOL measurements (the setup used for XEOL measurements has been earlier described in ref. (https://doi.org/10.1134/S0030400X22070086); #6 synthesis products collection flask; **(b)** #5 – silicon pipe entering the sample environment area from the outside of the lead cover; #7 – X-ray tube used for XEOL measurement; #8 – 3D-printed XEOL microfluidic chip equipped with rhombic-shaped reservoir filled with the synthesis product. Chip is fixed with adhesive tape on sample holder and located inside the sample chamber (small lead dome), so that the surface of the chip is tilted by 45° with respect to the X-ray beam and fluorescent detector window (see ref https://doi.org/10.1134/S0030400X22070086).

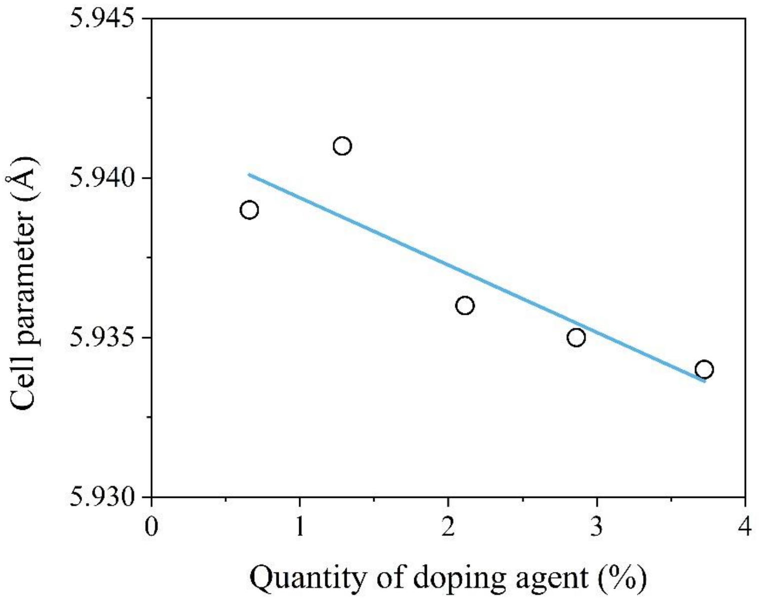

Figure S4. Correlation between the actual Tb^{3+} percentage and refined lattice parameter

Table S1. Calculated initial elemental composition of the $BaGd_{1-x}F_5{:}_xTb^{3+}$ samples and actual elemental composition from X-ray fluorescence measurements.

Sample names	Expected elemental composition at. %				Actual elemental composition at. %*				% of Gd^{3+} substitution by Tb^{3+}
	Ba	Gd	Tb	F	Ba	Gd	Tb	F	
5Tb	14,29	13,57	0,71	71,42	12,92	14,65	0,66	71,77	4,31
10Tb	14,29	12,86	1,43	71,42	13,02	13,95	1,29	71,74	8,44
15Tb	14,29	12,14	2,14	71,42	13,26	12,95	2,11	71,68	14,03
20Tb	14,29	11,43	2,86	71,42	12,99	12,39	2,86	71,76	18,76
25Tb	14,29	10,71	3,57	71,42	13,08	11,46	3,72	71,74	24,52

* Since, our XRF device is not capable of detecting elements with atomic mass lighter than sodium (Na), fluorine atoms (F) were added to the compound's formulas in an amount sufficient to completely compensate for all metals positive charges. The numerical proportions of the positively charged ions (Ba^{2+}, Gd^{3+} and Tb^{3+}) were recalculated to $5F^-$ anions. This method is widely used, for example, in mineralogy to compile mineral formulas.

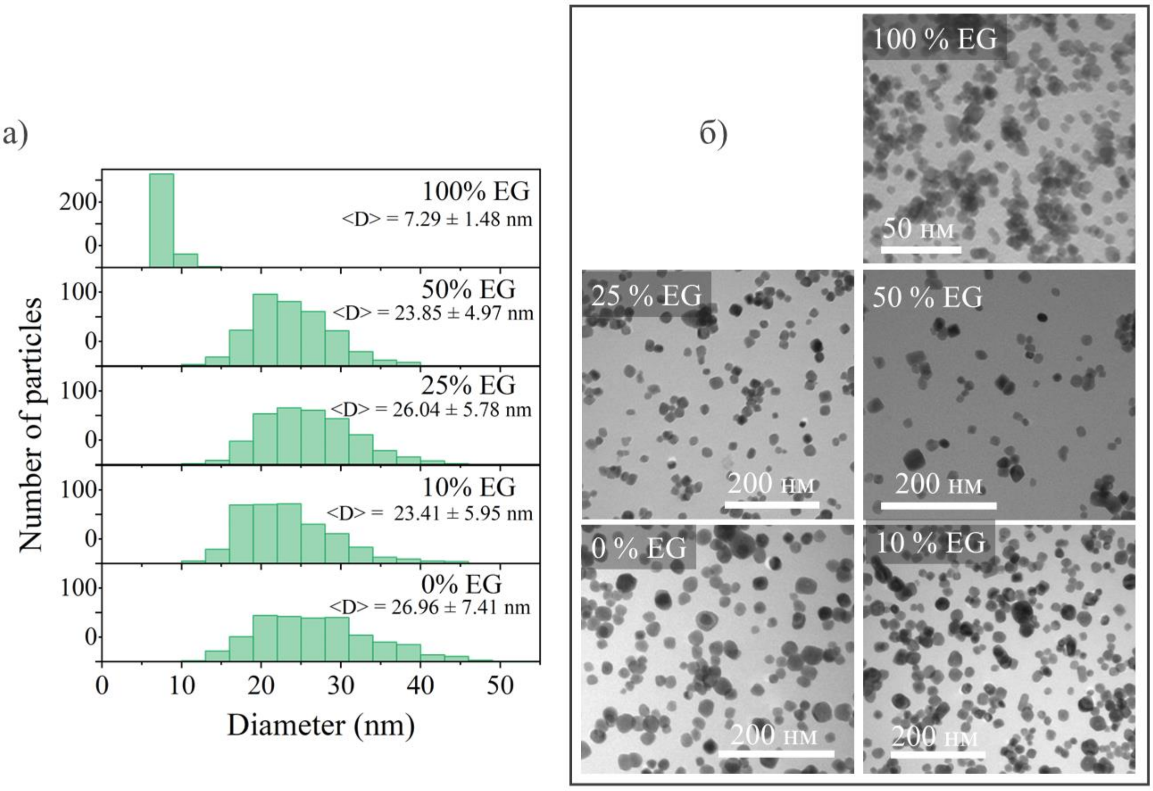

Figure S5. xTb samples, obtained by MW synthesis (**a**) particle size distribution and <D> with the mean size ± standard deviation according to the TEM; (**b**) TEM images. The figure was reproduced from our previous work [35].

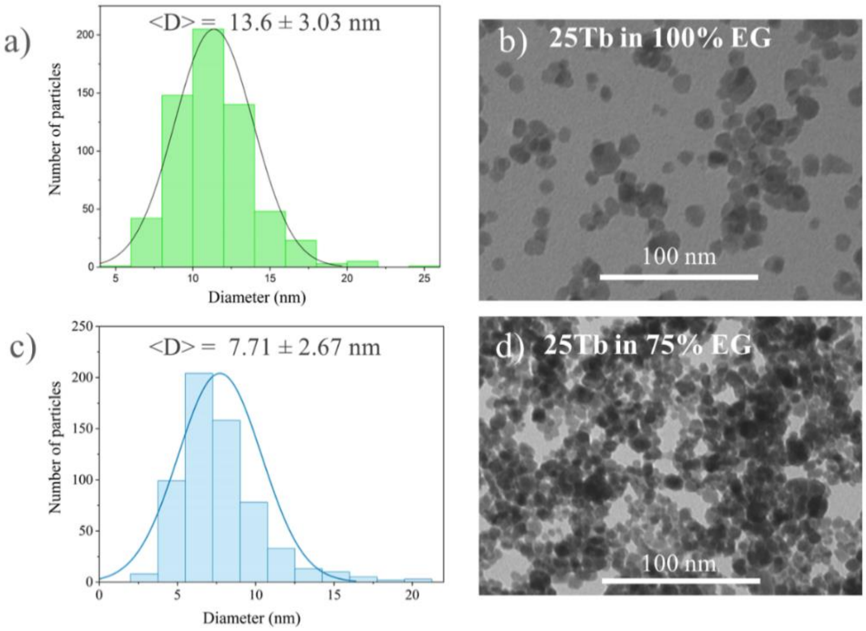

Figure S6. TEM images of 25Tb samples obtained in 100EG (**b**) and 75EG (**d**), and corresponding particle size distribution histograms (**a**, **c**).

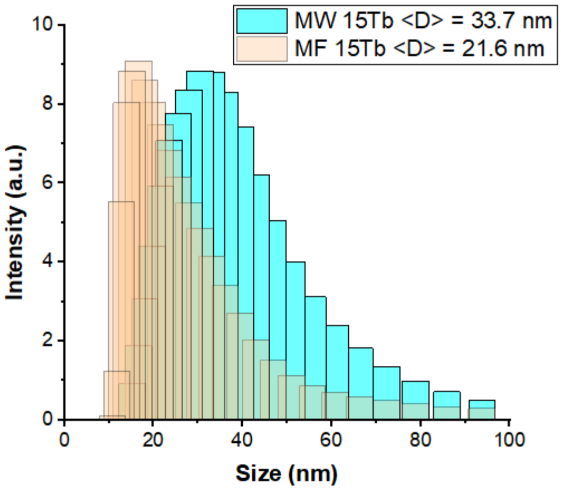

Figure S7. Nanoparticles size distribution curves and value of mean size as obtained from dynamic light scattering (DLS) measurements for $BaGdF_5$:15Tb sample obtained by MF and MW synthesis.

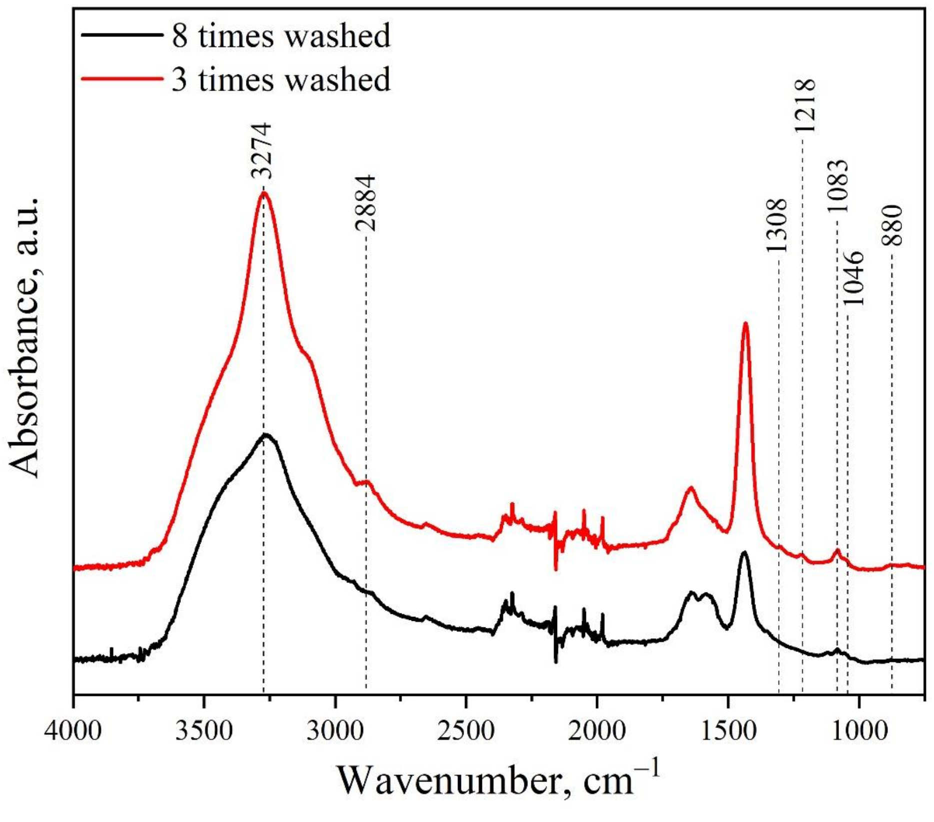

Figure S8. FTIR spectra of $BaGdF_5$:25Tb washed with DI water 3 and 8 times.

Absorption band at 880 cm^{-1} may be attributed to $\delta_{C\text{-}H}$ bonds of EG. The peaks at 1046 cm^{-1} and 1083 cm^{-1} belong to the coupling bands of C–O skeletal vibration modes and C–C stretching vibration modes. Band at 2884 cm^{-1} can be attributed to symmetrical stretching vibrations of the -CH_2- group. The -CH_2- group also has twisting and wagging vibrations at 1219 and 1306 cm^{-1},

respectively. After 8 times washing, a decrease in the intensity of vibrations of these bands is observed, associated with the partial removal of EG molecules from the particles surface. The same trend is observed for a broad peak at 3275 cm^{-1}, corresponding to vibrations of the OH-groups of water and EG molecules.

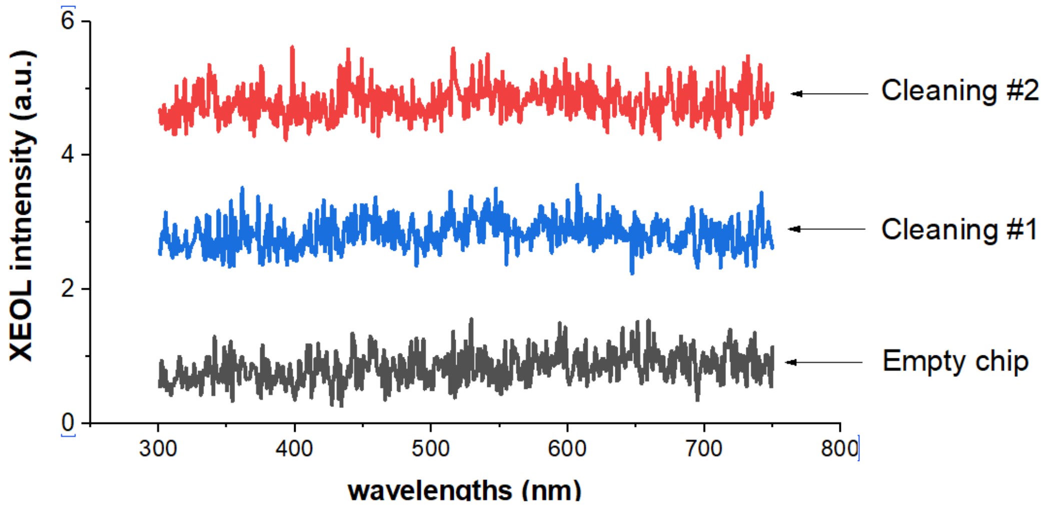

Figure S9. Estimation of the efficiency of cleaning procedure upon *in situ* XEOL acquisition for the series of $BaGd_{1-x}F_5:Tb_x$ synthesis with different amount of doping Tb element. Cleaning #1 signal was recorded upon cleaning between synthesis of 10Tb and 15Tb, while cleaning #2 – between synthesis 15Tb and 20Tb. In all cases the signal at the level of noise were obtained.

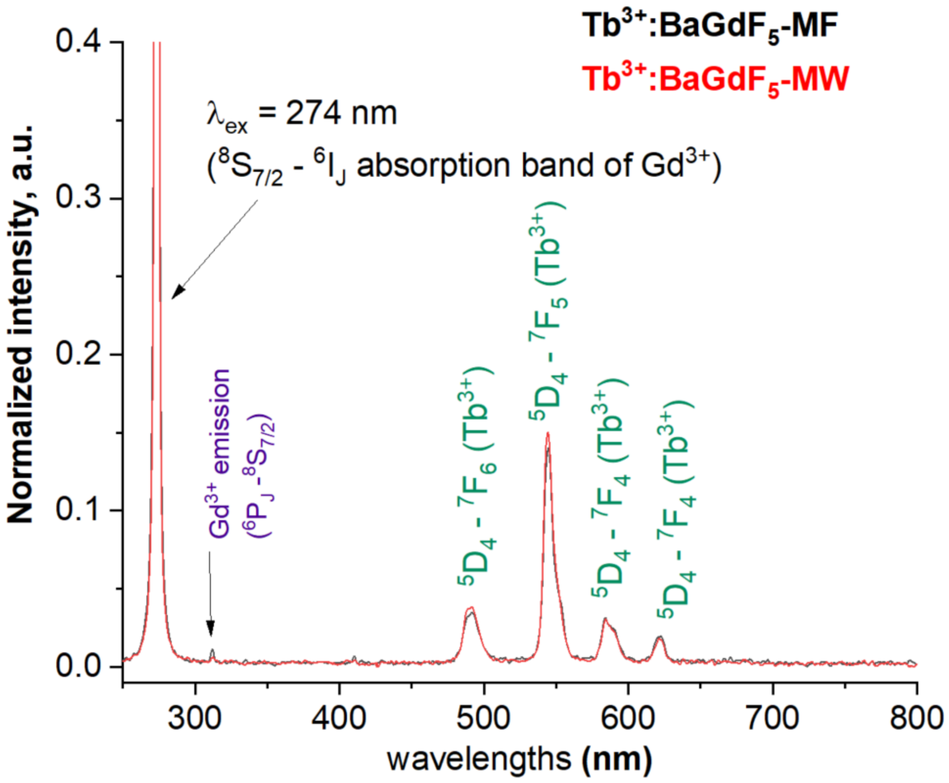

Figure S10. Comparison of the photoluminescent spectra collected for 15Tb samples synthesized by micro-wave (MW) and microfluidic (MF) approaches, upon direct excitation of Gd^{3+} ions (λ_{ex} = 274 nm, $^8S_{7/2} \rightarrow {}^6I_J$ adsorption band) accessed by the 3th harmonic of of LOTIS TII tunable laser LT-2211A (τ=10 ns, ν=10 Hz). Only tiny peak for Gd^{3+} emission line was observed, thus revealing an efficient $Gd^{3+} \rightarrow Tb^{3+}$ energy transfer.

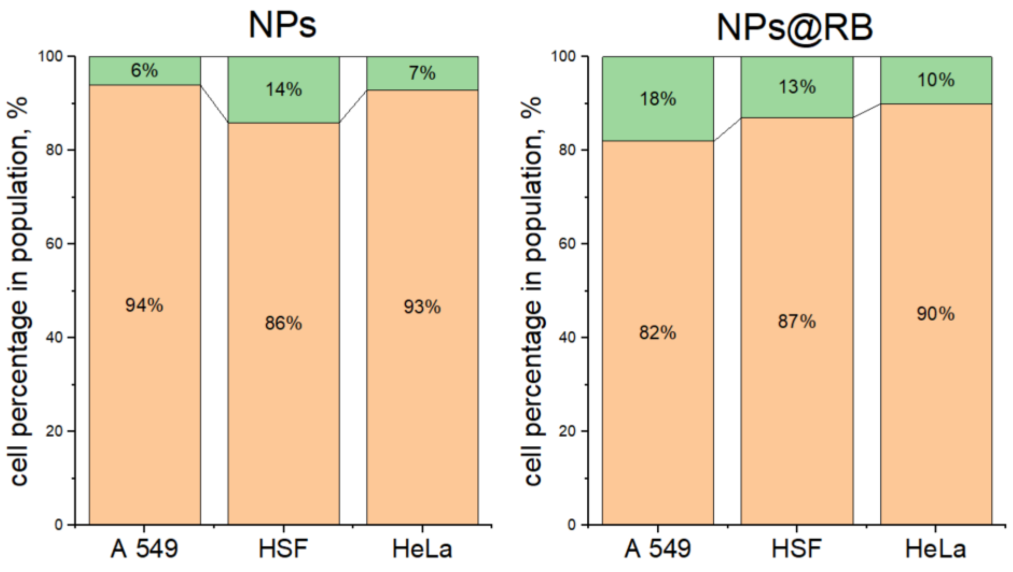

Figure S11. Cell viability estimation prior to flow cytometry experiments (orange – living cells, green – dead cells). Agent concentrations 400 μg/mL, incubation time 12 h.

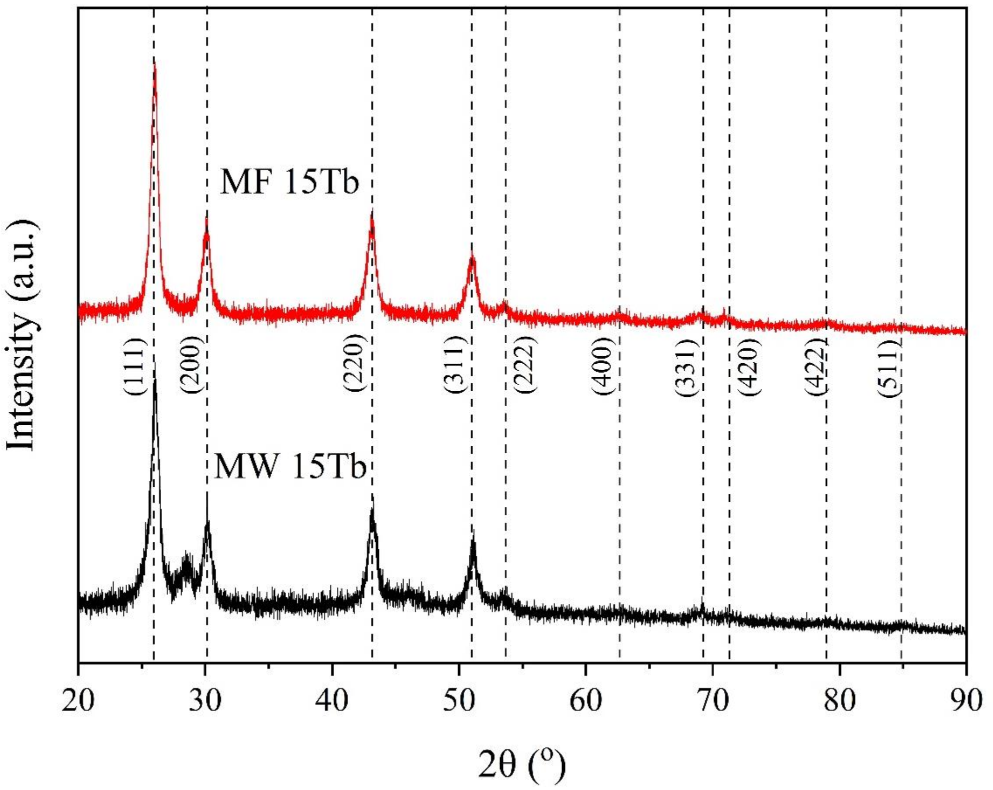

Figure S12. Comparison of 15Tb sample XRD profiles obtained by MF and MW methods.

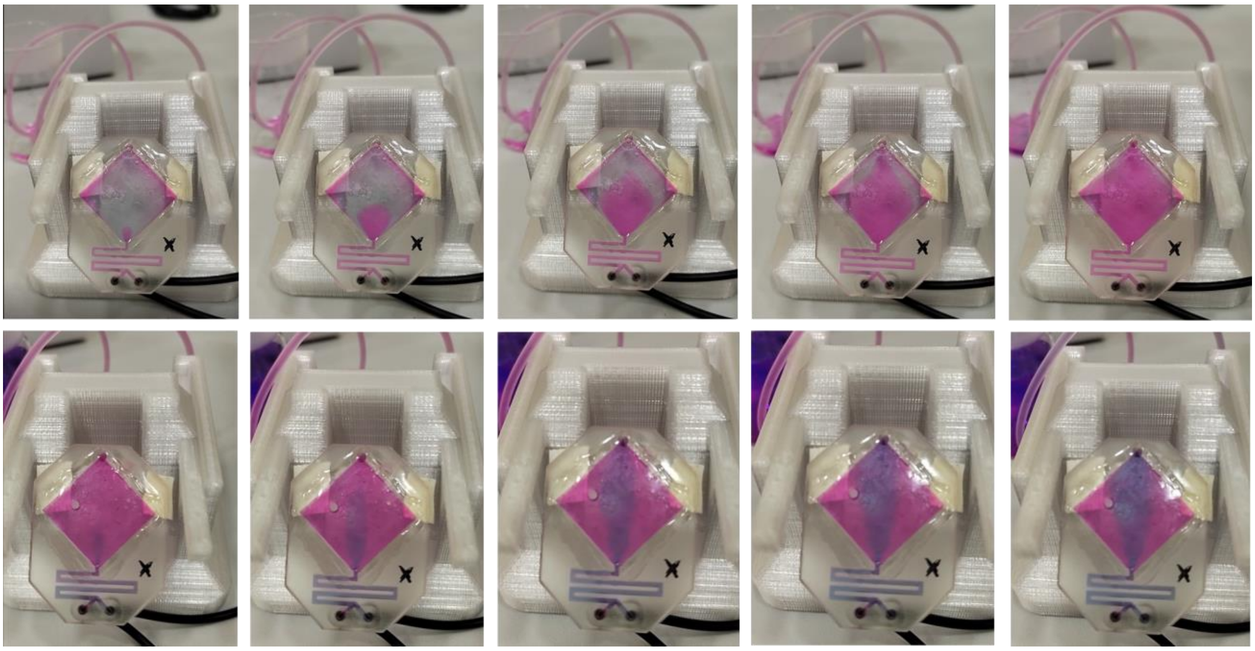

Figure S13. Visualization of liquid distribution in the reservoir of XEOL chip for the synthesis in 75 EG (top part) and aqueous (bottom part) solutions of Rose Bengal (red color) and Methylene blue (blue color) dyes.
